# Maternal geophagy in Ghana: a mini review of toxicological, microbial, nutritional, and radiological exposure pathways

**DOI:** 10.3389/fpubh.2026.1782963

**Published:** 2026-03-30

**Authors:** Henry Lawluvi, Cyrus Cyril Arwui, Ernest Sanyare Warmann Beinpuo, Augustine Faanu, Lilian Agyeman, Emmanuel Akrobortu, Theodora Amarh

**Affiliations:** 1 Nuclear Regulatory Authority, Accra, Ghana; 2 School of Nuclear and Allied Sciences, University of Ghana, Accra, Ghana; 3 Ghana Atomic Energy Commission, Accra, Ghana

**Keywords:** bioaccessibility, geophagy, Ghana, maternal–foetal health, microbial contamination, naturally occurring radionuclides, pregnancy, toxic metals

## Abstract

Maternal geophagy remains a common cultural and behavioural practice among pregnant women in Ghana, representing a potential pathway for various concurrent exposures, including toxicological, microbial, nutritional, and radiological. This Mini Review synthesises evidence from a wide range of multidisciplinary literature, encompassing mineralogy, toxicology, parasitology, microbiology, nutrition, and radiological science, to provide a comprehensive assessment of maternal geophagy in the Ghanaian context. The reviewed literature consistently indicates that geophagic clays contain elevated concentrations of toxic metals, such as lead, arsenic, cadmium, chromium, and mercury, which often exceed international safety limits. Bioaccessibility studies further demonstrate that metal solubility increases under gastric conditions, suggesting that actual maternal exposure may be underestimated when only considering total concentrations. In addition to chemical hazards, microbial contamination and the presence of geohelminths in commercially sold clays pose infection risks, exacerbated by informal extraction, processing, and handling practices. Emerging evidence specific to Ghana also shows that commonly consumed clays contain naturally occurring radionuclides, such as potassium-40, uranium-238, and thorium-232, which contribute to measurable internal doses that warrant closer examination, especially with repeated ingestion during pregnancy. Overall, these findings highlight maternal geophagy as a multi-hazard exposure pathway involving toxicological, microbial, nutritional, and radiological risks. Significant knowledge gaps remain, including the lack of bioavailability-adjusted radiological dose modelling and limited longitudinal evidence linking geophagy to foetal outcomes. This review highlights the importance of developing integrated exposure assessment frameworks, enhancing regulatory oversight, and implementing targeted public health interventions to address the multifaceted risks associated with maternal geophagy in Ghana.

## Introduction

Geophagy, the intentional consumption of earth materials such as clay or soil, is a long-standing practice observed in many cultures throughout history. In sub-Saharan Africa, this behaviour is particularly common among pregnant women. Geophagy has been interpreted not only as a culturally driven behaviour but also as a practice with potential physiological, nutritional, and psychosocial functions (1–6, 37). Certain clay-rich soils possess adsorptive properties that may bind toxins, pathogens, and excess gastric acid, which can temporarily relieve gastrointestinal discomfort (1, 3, 5, 6).

Geophagic materials may also contain trace minerals, although their nutritional contribution is often limited due to low or variable bioavailability and, in some cases, interference with nutrient absorption (1, 2, 5–7). In addition, geophagy plays important psychosocial roles, including coping with pregnancy-related discomfort, reinforcing cultural identity, and maintaining social belonging (8–12).

Despite these proposed benefits, the magnitude and consistency of such effects remain uncertain, and available evidence indicates that potential advantages must be weighed against well-documented risks, including heavy metal exposure, parasitic infection, and nutritional impairment (1–3, 6, 13–18). While geophagy is frequently regarded as harmless or beneficial, increasing multidisciplinary research suggests it represents a significant environmental exposure pathway, with implications for maternal and foetal health.

Evidence from multiple regions of Africa, including West Africa (Ghana, Nigeria), East Africa (Tanzania, Kenya, Uganda), and Southern Africa (South Africa, Namibia), indicates that geophagic practices are widespread and associated with measurable health risks (1, 14, 19–21). Geochemical and toxicological studies conducted in Ghana and similar African contexts consistently report high concentrations of toxic metals, including lead, arsenic, cadmium, chromium, and mercury, in geophagic clays, often exceeding international safety thresholds (22–24). Bioaccessibility studies further demonstrate that these metals become more soluble under gastric conditions, indicating that total concentration measurements may underestimate actual maternal exposure and the potential for placental transfer (20). Beyond chemical toxicity, infectious and parasitological risks add further complexity to the health profile associated with maternal geophagy. Studies have documented the presence of geohelminths, bacteria, and fungal contaminants in market-sold clays and materials sourced from informal extraction sites, often resulting from poor handling, storage, and processing practices (22, 25). Additionally, the mineralogical properties of commonly consumed kaolinite-rich clays may hinder the absorption of essential micronutrients such as iron and zinc, exacerbating pregnancy-related anaemia and nutritional deficiencies (14).

Evidence from multiple regions of Africa, including West Africa (Ghana, Nigeria), East Africa (Tanzania, Kenya, Uganda), and Southern Africa (South Africa, Namibia), indicates that geophagic practices are widespread and associated with measurable health risks (1, 14, 19–21). Geochemical and toxicological studies conducted in Ghana and similar African contexts consistently report high concentrations of toxic metals, including lead, arsenic, cadmium, chromium, and mercury, in geophagic clays, often exceeding international safety thresholds (22–24). Bioaccessibility studies further demonstrate that these metals become more soluble under gastric conditions, indicating that total concentration measurements may underestimate actual maternal exposure and the potential for placental transfer (20).

Another, albeit underexplored, aspect of maternal geophagy is the potential for radiological exposure. Evidence from several African regions indicates that clays and soils consumed for geophagy contain naturally occurring radionuclides, particularly potassium-40, uranium-238, and thorium-232, linked to regional geology and mineralisation processes (26). While specific data for Ghana remain limited, recent assessments suggest that chronic ingestion of these materials may result in measurable internal doses, raising concerns about cumulative exposure during pregnancy and its potential effects on the foetus (20).

Despite the expanding body of literature, research on maternal geophagy remains fragmented across disciplines, with few studies taking an integrated approach that considers toxicological, microbial, nutritional, and radiological pathways simultaneously. In Ghana, regulatory oversight of clay extraction, processing, and sale is minimal, and antenatal care systems do not routinely screen for geophagy-related exposures (22). Additionally, there is a significant lack of bioavailability-adjusted radiological dose modelling, as well as limited longitudinal evidence linking maternal geophagy to foetal and early-life outcomes.

In light of this context, this Mini Review synthesizes current evidence on the prevalence, driving factors, and multi-hazard health risks associated with maternal geophagy in Ghana. By integrating findings from geochemistry, toxicology, microbiology, nutrition, and radiological science, the review aims to clarify key exposure pathways, identify critical knowledge and policy gaps, and highlight priorities for research, regulation, and public health intervention within the realm of maternal and reproductive health.

## Methodology

This Mini Review adopts an integrative narrative approach to synthesise multidisciplinary evidence on maternal geophagy and its associated health risks in Ghana. This method was chosen to consolidate a diverse body of literature from behavioural science, geochemistry, toxicology, microbiology, nutrition, and radiological assessment. While these studies cannot be effectively combined through traditional meta-analysis, they remain highly relevant for understanding reproductive health.

The literature search was conducted across major academic databases, including PubMed, Web of Science, Scopus, ScienceDirect, SpringerLink, Google Scholar, and African Journals Online (AJOL). Additionally, targeted searches were performed using repositories from the World Health Organisation, Food and Agriculture Organisation, International Atomic Energy Agency, and the United Nations Scientific Committee on the Effects of Atomic Radiation. The searches covered publications up to March 2025 and utilized various combinations of keywords related to geophagy, pregnancy, toxic metals, bioaccessibility, microbial contamination, and naturally occurring radionuclides.

Studies were included if they examined geophagy or pica among pregnant women; characterised the mineralogical, geochemical, microbial, or radiological composition of geophagic clays; assessed exposure to toxic metals or bioaccessible fractions; or reported maternal or foetal health outcomes relevant to geophagy. While evidence from Ghana was prioritized, studies from comparable African contexts were included to support interpretation where Ghana-specific data were limited. Animal-only studies, non-empirical commentaries, and publications lacking methodological clarity were excluded.

Data extraction focused on key thematic areas instead of comprehensive quantitative comparisons. The included studies were examined for information on exposure pathways, contaminant profiles, biological plausibility, and health implications relevant to maternal–foetal outcomes. Findings were synthesized narratively to identify converging evidence, recurring risks, and significant knowledge gaps, with particular emphasis on the interactions of chemical, microbial, nutritional, and radiological hazards.

### Evidence synthesis: maternal geophagy and multi-hazard health risks

#### Prevalence and drivers of maternal geophagy in Ghana

Maternal geophagy, the practice of consuming clay or soil, is widely accepted and practised in Ghana, spanning both rural and urban areas. Research shows that a significant number of pregnant women engage in this behaviour, often starting in the first or second trimester and continuing intermittently throughout their pregnancy (22, 27). Although estimates of how common this practice is vary depending on the location and study methods, its persistence reflects deeply rooted cultural norms, sensory preferences, and physiological discomforts associated with pregnancy.

The reasons behind geophagy are complex. Many women report that consuming clay provides relief from symptoms such as nausea, excessive salivation, heartburn, and changes in appetite. Additionally, it offers emotional comfort and aligns with culturally held beliefs about foetal well-being (14, 19). This practice is not restricted to women with limited formal education or those living in rural areas; studies conducted in Ghana and among African migrant populations indicate that geophagy continues even among women who are aware of the potential health risks (8, 28, 33). This underscores the limitations of purely biomedical explanations and emphasises the need to understand geophagy as both a behavioural and socio-cultural phenomenon.

#### Chemical and nutritional hazards of geophagic clays

A consistent finding in studies conducted in Ghana and the surrounding region is the presence of elevated levels of toxic metals in geophagic clays. Analyses of clays consumed by pregnant women in Ghana indicate that levels of lead, arsenic, cadmium, chromium, and manganese often exceed international safety thresholds (17, 22, 24, 35). These contaminants arise from both natural geological mineralisation and human activities, such as artisanal mining, quarrying, and environmental pollution.

Importantly, bioaccessibility studies show that metals bound within clay matrices can become more soluble under gastric conditions, which increases the likelihood of systemic absorption during digestion (20). This finding challenges exposure assessments that rely solely on total elemental concentrations and suggests that the risks to maternal and fetal health may be underestimated in conventional evaluations. Toxic metals like lead and mercury are well-known neurotoxicants, while arsenic and cadmium have been associated with placental dysfunction, fetal growth restriction, and impaired immune development (23, 29).

Additionally, the mineralogical properties of commonly consumed kaolinite-rich clays can interfere with the absorption of essential micronutrients. The high cation-exchange capacity of these clays can bind dietary iron, zinc, and calcium, exacerbating micronutrient deficiencies and contributing to the high prevalence of anaemia among geophagic pregnant women (14, 30). These nutritional interactions represent a critical but often overlooked pathway linking geophagy to adverse pregnancy outcomes.

#### Microbial and parasitological exposure pathways

In addition to chemical hazards, geophagic materials may serve as vectors for infectious agents. Studies conducted in Ghana and other African settings have identified bacterial contamination, fungal growth, and geohelminth eggs in clays sold in informal markets (22, 25). Contamination is frequently associated with informal extraction, roadside drying, inadequate storage, and poor market hygiene.

Parasitological exposure is of particular concern in pregnancy, as helminth infections contribute to chronic blood loss, inflammation, and anaemia, compounding the nutritional vulnerabilities already present in many pregnant women (31). While some processing methods may reduce microbial viability, evidence suggests that contamination risks are not fully eliminated, especially in commercially distributed clays lacking quality control (9). These findings reinforce the need to consider infectious hazards alongside chemical toxicity when assessing the health implications of maternal geophagy. In addition to chemical hazards, geophagic materials may act as carriers for infectious agents. Studies conducted in Ghana and other African regions have found bacterial contamination, fungal growth, and geohelminth eggs in clays sold in informal markets (22, 25). Contamination is often linked to informal extraction methods, roadside drying, inadequate storage, and poor market hygiene.

Parasitological exposure is particularly concerning during pregnancy, as helminth infections can lead to chronic blood loss, inflammation, and anaemia, worsening the nutritional vulnerabilities that many pregnant women already face (31, 34). Although some processing methods may reduce microbial viability, evidence indicates that the risks of contamination are not eliminated, especially in commercially distributed clays that lack quality control (9). These findings highlight the importance of considering infectious hazards alongside chemical toxicity when evaluating the health implications of maternal geophagy.

### Emerging radiological considerations and multi-hazard interactions

Radiological exposure is one of the least studied, yet potentially significant, aspects of maternal geophagy. Research conducted in various African contexts has shown that geophagic clays contain naturally occurring radionuclides such as potassium-40, uranium-238, thorium-232, and radium-226, which are reflective of the geological formations in the area (26, 32). Chronic ingestion of these materials may lead to internal radiation doses, especially when consumed repeatedly during pregnancy.

While specific radiological data on geophagic clays in Ghana are limited, existing evidence indicates that the internal doses from ingestion could be substantial under habitual consumption scenarios (20). One significant gap in current exposure science is the lack of bioaccessibility-adjusted dose modelling and foetal dose estimation. When considered alongside toxic metal exposure, micronutrient depletion, and microbial infection, radiological ingestion adds to a multilayered exposure profile that could impact placental function and foetal development.

**Figure 1:**
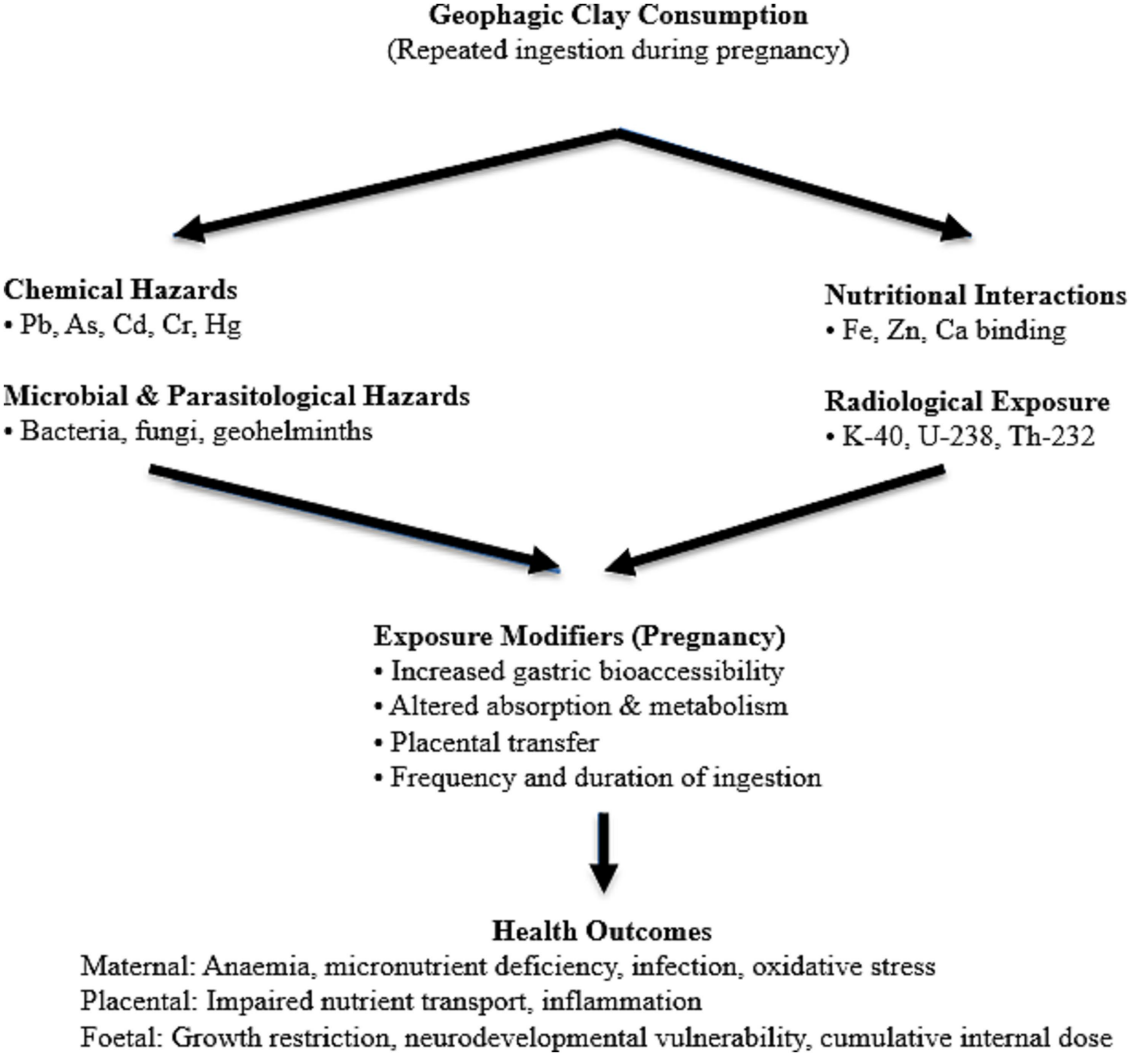
Integrated multi-hazard exposure pathway associated with maternal geophagy in Ghana. The schematic illustrates how geophagic clay consumption during pregnancy can result in concurrent exposure to toxic metals, microbial and parasitological agents, micronutrient-binding mineral phases, and naturally occurring radionuclides. These exposure pathways operate in parallel and are modified by pregnancy-related physiological factors, including increased bioaccessibility and placental transfer, leading to cumulative maternal, placental, and fetal health risks. The framework conceptualizes maternal geophagy as a multidimensional environmental exposure rather than a single-pathway behaviour.

The main hazard categories, exposure routes, and their associated maternal–foetal health implications linked to geophagy in Ghana are summarised in Table 1, while their integrated and interacting exposure pathways are illustrated in Figure 1.

**Table 1:** Major hazard categories and health implications associated with maternal geophagy in Ghana.

Hazard category	Representative agents	Primary exposure route	Key maternal implications	Potential foetal implications
Toxic metals	Pb, As, Cd, Cr, Hg	Oral ingestion; increased gastric bioaccessibility	Anaemia, oxidative stress, endocrine disruption	Growth restriction, neurodevelopmental vulnerability
Nutritional interactions	Kaolinite-rich clays binding Fe, Zn, Ca	Reduced dietary absorption	Micronutrient deficiency, iron-deficiency anaemia	Impaired growth, altered developmental programming
Microbial contamination	Bacteria, fungi	Oral ingestion from contaminated clays	Gastrointestinal infection, inflammation	Indirect effects via maternal illness
Parasitological exposure	Geohelminths (e.g., *Ascaris*, hookworm)	Oral ingestion	Chronic blood loss, inflammation	Reduced oxygen and nutrient delivery
Radiological exposure (NORMs)	^40^K, ^238^U, ^232^Th	Chronic ingestion	Low-level internal dose accumulation	Uncertain foetal dose; radiosensitive period

The table summarises key chemical, nutritional, microbial, parasitological, and radiological hazards linked to geophagic clay consumption during pregnancy, highlighting exposure routes and potential maternal and foetal health effects. The emphasis is on integrated risk pathways rather than individual contaminant concentrations.

Overall, the convergence of chemical, microbial, nutritional, and radiological pathways presents maternal geophagy as a complex environmental health issue rather than merely a behaviour associated with a single risk. Existing studies often fail to integrate these hazards within a comprehensive framework, which hinders the assessment of cumulative risk and undermines the development of effective regulatory and public health responses.

## Discussion

This review examines maternal geophagy in Ghana as a complex environmental health behaviour situated at the intersection of cultural practices, nutritional vulnerability, and exposure to multiple hazards. While many pregnant women view geophagy as beneficial or harmless, the evidence indicates that it may pose significant and often under-recognised risks related to toxicological, microbial, nutritional, and possibly radiological factors. Importantly, these risks do not operate independently; rather, they interact in ways that can exacerbate adverse outcomes for both mothers and their babies.

A key insight from this synthesis is that while chemical toxicity is crucial, it is not sufficient on its own to explain the health implications of geophagy. Elevated levels of toxic metals such as lead, arsenic, cadmium, and chromium have been repeatedly documented in geophagic clays consumed in Ghana and similar African contexts (22–24, 36). Bioaccessibility studies indicate that gastric conditions can increase metal solubility, suggesting that conventional assessments based solely on total concentrations may underestimate biologically relevant exposure (20). These findings are particularly alarming during pregnancy, where the placental transfer of metals and the foetal susceptibility to oxidative stress and neurotoxicity are well established (29).

In addition to chemical exposure, the nutritional and infectious aspects of geophagy seem to worsen maternal vulnerability. The cation-exchange properties of kaolinite-rich clays can hinder the absorption of iron and zinc, leading to anaemia and micronutrient deficiencies that are already common among pregnant women in Ghana (14, 30). At the same time, microbial and parasitological contamination of commercially sold clays introduces further pathways for inflammation, blood loss, and compromised immune function (25, 31). The presence of these multiple mechanisms suggests that the adverse outcomes associated with geophagy may result from cumulative effects rather than single-agent exposures.

One important yet often overlooked finding is the possible contribution of radiological exposure to the overall risk associated with maternal geophagy. Evidence from African contexts shows that geophagic clays may contain naturally occurring radionuclides, including potassium-40, uranium-238, and thorium-232, which could contribute to internal doses in scenarios of chronic ingestion (26, 32). Although current data indicate that radiation doses may fall within public exposure limits in many areas, pregnancy constitutes a radiosensitive period. Additionally, the absence of bioavailability-adjusted ingestion models and foetal dose estimates creates significant uncertainty (20). When considering chemical toxicity and nutritional stress alongside radiological exposure, even low-level chronic exposure to radiation deserves closer examination.

The persistence of geophagy, despite its documented risks, highlights the limitations of risk communication strategies that characterise the practice solely as harmful. Cultural norms, sensory preferences, emotional comfort, and unmet nutritional needs play significant roles in shaping women’s engagement with geophagy, even among those who are aware of potential dangers (14, 19). This suggests that purely prohibitive or alarmist messaging may be ineffective and could undermine trust in antenatal care services. Instead, culturally sensitive counselling that acknowledges women’s motivations while addressing the associated risks is more likely to lead to meaningful behaviour change.

From a regulatory and policy perspective, this review highlights significant gaps in Ghana’s environmental and maternal health governance. The extraction, processing, and sale of geophagic clays are largely unregulated, and routine screening for pica or clay consumption is not incorporated into antenatal care protocols or broader maternal health policy frameworks (22). Additionally, environmental monitoring frameworks seldom consider ingestion pathways for naturally occurring radionuclides, focusing instead on occupational or external exposure routes. These gaps hinder health and regulatory authorities in identifying, monitoring, and mitigating cumulative exposure risks affecting pregnant women, while undermining the integration of environmental risk considerations into maternal health programs.

Several critical research needs emerge from this synthesis. There is a need for integrated multi-hazard studies that simultaneously assess toxic metals, microbial contamination, nutrient interactions, and radionuclide content in geophagic materials. It is necessary to develop Ghana-specific bioaccessibility and ingestion-based dose models, including foetal dose estimation, which is essential for refining risk characterisation for pregnancy. Longitudinal studies that link maternal geophagy to birth outcomes and early childhood development would strengthen causal inferences and inform targeted interventions. Finally, supply-chain analyses that trace contamination points from extraction to market could support evidence-based regulation of geophagic clay products.

Overall, this review reframes maternal geophagy in Ghana not as a singular risk or purely cultural practice, but as a multi-dimensional exposure pathway influenced by geological, biological, social, and regulatory factors. Addressing maternal geophagy health implications requires interdisciplinary research, coordinated policy action, and antenatal interventions that balance cultural sensitivity with evidence-based risk reduction.

## Conclusion

Maternal geophagy in Ghana is a complex practice that involves cultural traditions, environmental influences, and reproductive health risks. While this behaviour is strongly ingrained in social norms and often viewed as beneficial during pregnancy, the evidence in this review shows that consuming clay can expose women and their unborn babies to various hazards. These include toxic metals, decreased absorption of essential micronutrients, microbial and parasitological contamination, and possible internal exposure to naturally occurring radionuclides. The cumulative impact of these exposures, especially with repeated ingestion throughout pregnancy, emphasises the need for more thorough risk assessments that account for multiple exposure pathways.

The review points out that current approaches to maternal geophagy in Ghana are fragmented, lacking integration across toxicology, nutrition, microbiology, and radiology. Oversight of clay extraction and sales is minimal, and antenatal care systems typically do not treat geophagy as a significant exposure risk. As a result, health risks and opportunities for early intervention are frequently ignored.

To effectively address maternal geophagy, a coordinated response is essential. This should combine interdisciplinary research, targeted public health strategies, and culturally sensitive antenatal counselling. Steps such as strengthening environmental monitoring, incorporating geophagy screening into maternal health services, and developing integrated exposure assessment frameworks are crucial for reducing avoidable risks while respecting the cultural contexts in which this practice exists. Ultimately, reframing maternal geophagy as a multifaceted environmental health issue can foster more balanced, evidence-based policies that aim to protect maternal and foetal health in Ghana.

## References

[1] Bonglaisin J Kunsoan NB Bonny P Matchawe C Tata BN Nkeunen G . Geophagia: benefits and potential toxicity to humans. Front Public Health. (2022) 10:893831. doi: 10.3389/fpubh.2022.893831, 35958861 PMC9360771

[2] Frazzoli C Pouokam G Mantovani A Orisakwe O. Health risks from lost awareness of cultural behaviours rooted in traditional medicine: an insight in geophagy and mineral intake. Sci Total Environ. (2016) 566-567:1465–71. doi: 10.1016/j.scitotenv.2016.06.028, 27342642

[3] Gomes C. Healing and edible clays: a review of basic concepts, benefits and risks. Environ Geochem Health. (2018) 40:1739–65. doi: 10.1007/s10653-016-9903-4, 28150053

[4] Gomes C Rautureau M Poustis J Gomes J. Benefits and risks of clays and CLAY minerals to human health from ancestral to current times: a synoptic overview. Clay Clay Miner. (2021) 69:612–32. doi: 10.1007/s42860-021-00160-7

[5] Reid R. Cultural and medical perspectives on geophagia. Med Anthropol. (1992) 13:337–51. doi: 10.1080/01459740.1992.9966056, 1545692

[6] Young S Miller J. Medicine beneath your feet: a biocultural examination of the risks and benefits of geophagy. Clay Clay Miner. (2019) 67:81–90. doi: 10.1007/s42860-018-0004-6

[7] Malepe R Candeias C Mouri H. Geophagy and its potential human health implications—a review of some cases from South Africa. J Afr Earth Sci. (2023) 200:104848. doi: 10.1016/j.jafrearsci.2023.104848

[8] Madziva C Chinouya M Njoroge K. Experiences of geophagy during pregnancy among African migrant women in London: implications for public health interventions. SSM Qual Res Health. (2024) 5:100431. doi: 10.1016/j.ssmqr.2024.100431

[9] Mafe AN Makinde O Adeleke RA. Geophagia among pregnant women: microbiological and toxicological safety implications. Environ Geochem Health. (2025) 47:347. doi: 10.1007/s10653-025-02656-w, 40736606 PMC12310767

[10] Singh L. Geophagy in Manipur: cultural practices and medical geology perspectives. Int J Sci Res. (2025) 14:1047–51. doi: 10.21275/sr25614121102

[11] Hunter J DeKleine R. Geophagy in Central America. Geogr Rev. (1984) 74:157–69. doi: 10.2307/214097, 11614511

[12] Malebatja M Randa M Mokgatle M Oguntibeju O. Health education and promotion interventions to mitigate geophagic practise: a scoping review. Public Health Rev. (2025) 46:1607614. doi: 10.3389/phrs.2025.1607614, 40371320 PMC12074957

[13] Ali A. Potential source of heavy metals in the geophagic clay (marl) and its implication on human health in NE Iraq: a pilot study. Iraqi Geol J. (2021) 54:80–7. doi: 10.46717/igj.54.2c.8ms-2021-09-27

[14] Kambunga S Candeias C Hasheela I Mouri H. Review of the nature of some geophagic materials and their potential health effects on pregnant women: some examples from Africa. Environ Geochem Health. (2019) 41:2949–75. doi: 10.1007/s10653-019-00288-5, 30977022

[15] Malebatja M Randa M Mokgatle M Oguntibeju O. Chemical composition of clay soil analysis and potential health risks: experimental study in Tshwane District, Gauteng Province. Appl Sci. (2024) 14:9152. doi: 10.3390/app14199152

[16] Molale T Ebouel F Eze P. Beyond consumption: a multi-pathway human health exposure risk assessment of potentially toxic elements in geophagic soils of Botswana. Environ Geochem Health. (2025) 47:471. doi: 10.1007/s10653-025-02791-4, 41028598

[17] Orisakwe OE Udowelle NA Azuonwu O Nkeiruka IZ Nkereuwem UA Frazzoli C. Cadmium and lead in geophagic clay consumed in Southern Nigeria. Environ Geochem Health. (2020) 42:3865–75. doi: 10.1007/s10653-020-00632-0, 32607698

[18] Rukondo CE Mgina CA Pratap HB. Mineral composition and heavy metal risk assessment of geophagic soils from Tanzania. Toxicol Rep. (2024) 12:534–41. doi: 10.1016/j.toxrep.2024.04.008, 38778800 PMC11108962

[19] Davies T. Current status of research and gaps in knowledge of geophagic practices in Africa. Front Nutr. (2023) 9:1084589. doi: 10.3389/fnut.2022.1084589, 36890865 PMC9987423

[20] Kutalek R Wewalka G Gundacker C . Geophagy and potential health implications: geohelminths, microbes and heavy metals. Transactions of the Royal Society of Tropical Medicine and Hygiene. (2010) 104:787–795. doi: 10.1016/j.trstmh.2010.09.002 20889178

[21] Njiru H Elchalal U Paltiel O. Geophagy during pregnancy in Africa: a literature review. Obstet Gynecol Surv. (2011) 66:452–9. doi: 10.1097/ogx.0b013e318232a034 21944157

[22] Mensah FO Twumasi P Amenawonyo XK Larbie C Jnr AK. Pica practice among pregnant women in the Kumasi metropolis of Ghana. Int Health. (2010) 2:282–6. doi: 10.1016/j.inhe.2010.09.004, 24037870

[23] Decaudin P Kanagaratnam L Kmiec I Nguyen Y Migault C Lebrun D . Prevalence of geophagy and knowledge about its health effects among native Sub-Saharan Africa, Caribbean and South America healthy adults living in France. Eat Weight Disord. (2020) 25:465–469. doi: 10.1007/s40519-018-0624-9, 30547293

[24] Nkansah MA Korankye M Darko G Dodd M. Heavy metal content and health risk of geophagic white clay in Ghana. Toxicol Rep. (2016) 3:644–51. doi: 10.1016/j.toxrep.2016.08.005, 28959588 PMC5616015

[25] Kutalek R Wewalka G Gundacker C Auer H Wilson J Haluza D . Geophagy and potential health implications. Trans R Soc Trop Med Hyg. (2010) 104:787–95. doi: 10.1016/j.trstmh.2010.09.002, 20889178

[26] Ekosse G Nkeng G Bukalo N Oyebanjo O. Geophagic clays from Cameroon: provenance, metal contamination and health risk assessment. Int J Environ Res Public Health. (2021) 18:8315. doi: 10.3390/ijerph18168315, 34444064 PMC8394028

[27] Mensah F Twumasi P Amenawonyo XK Larbie C Baffo Jnr AK. Pica practice among pregnant women in the Kumasi metropolis of Ghana. Int Health. (2010) 2:282–6. doi: 10.1016/j.inhe.2010.09.004 24037870

[28] Decaudin P Kanagaratnam L Kmiec I Nguyen Y Migault C Lebrun D . Prevalence of geophagy and knowledge about its health effects among African migrant populations in France. Eat Weight Disord. (2018) 25:465–9. doi: 10.1007/s40519-018-0624-9 30547293

[29] Miller JD Collins SM Omotayo M Martin SL Dickin KL Young SL. Geophagic earths consumed by women in western Kenya contain dangerous levels of lead, arsenic, and iron. Am J Hum Biol. (2018) 30:e23118. doi: 10.1002/ajhb.23130, 29722093 PMC6105564

[30] Tayie F Koduah G Mork S. Geophagia clay soil as a source of mineral nutrients and toxicants. Afr J Food Agric Nutr Dev. (2013) 13:7087–100. doi: 10.18697/ajfand.56.12580

[31] Mouri H Malepe RE Candeias C. Geochemical composition and potential health risks of geophagic materials: an example from a rural area in the Limpopo Province of South Africa. Environ Geochem Health. (2023) 45:6305–6322. doi: 10.1007/s10653-023-01551-6, 37296282 PMC10403411

[32] Young SL Sherman PW Lucks JB Pelto GH Rowe L. Why On Earth?: Evaluating Hypotheses About The Physiological Functions Of Human Geophagy. The Quarterly Review of Biology. (2011) 86:97–120. doi: 10.1086/659884, 21800636

[33] Caillet P Poirier M Grall-Bronnec M Marchal E Pineau A Pintas C . High prevalence of kaolin consumption in migrant women living in a major urban area of France: A cross-sectional investigation. PLoS One. (2019) 14:e0219545. doi: 10.1371/journal.pone.0220557, 31365572 PMC6668907

[34] Ivoke N Ikpor N Ivoke O Ekeh F Ezenwaji N Odo G . Geophagy as risk behaviour for gastrointestinal nematode infections among pregnant women attending antenatal clinics in a humid tropical zone of Nigeria. Afr Health Sci. (2017) 17:24–31. doi: 10.4314/ahs.v17i1.5, 29026374 PMC5636248

[35] Kortei NK Koryo-Dabrah A Akonor PT Manaphraim NYB Ayim-Akonor M Boadi NO . Health risk assessment of toxic metals in geophagic clay consumed by pregnant women in Ghana. BMC Pregnancy Childbirth. (2020) 20:160. doi: 10.1186/s12884-020-02857-4, 32169034 PMC7071753

[36] Mouri H Malepe R Candeias C. Geochemical composition and potential health risks of geophagic materials: an example from a rural area in the Limpopo Province of South Africa. Environ Geochem Health. (2023) 45:6305–22. doi: 10.1007/s10653-023-01551-6, 37296282 PMC10403411

[37] Young SL Sherman PW Lucks JB Pelto GH. Why on earth? Evaluating hypotheses about human geophagy. Q Rev Biol. (2011) 86:97–120. doi: 10.1086/659884, 21800636

